# Subtype-Based Analysis of Cell-in-Cell Structures in Esophageal Squamous Cell Carcinoma

**DOI:** 10.3389/fonc.2021.670051

**Published:** 2021-06-11

**Authors:** Yuqi Wang, Zubiao Niu, Lulin Zhou, Yongan Zhou, Qunfeng Ma, Yichao Zhu, Mengzhe Liu, Yinan Shi, Yanhong Tai, Qiuju Shao, Jianlin Ge, Jilei Hua, Lihua Gao, Hongyan Huang, Hong Jiang, Qiang Sun

**Affiliations:** ^1^ College of Life Science and Bioengineering, School of Science, Beijing Jiaotong University, Beijing, China; ^2^ Research Unit of Cell Death Mechanism, Institute of Biotechnology, Chinese Academy of Medical Science, Beijing, China; ^3^ School of Medicine, Nankai University, Tianjin, China; ^4^ Department of Thoracic Surgery, The Second Affiliated Hospital of Air Force Military Medical University, Xi’an, China; ^5^ Department of Thoracic Surgery, The Fifth Medical Center of Chinese PLA General Hospital, Beijing, China; ^6^ Institute of Acupuncture and Moxibustion, China Academy of Chinese Medical Sciences, Beijing, China; ^7^ Department of Pathology, The Fifth Medical Center of Chinese PLA General Hospital, Beijing, China; ^8^ Department of Radiotherapy, The Second Affiliated Hospital of Air Force Military Medical University, Xi’an, China; ^9^ Department of Oncology, Beijing Shijitan Hospital of Capital Medical University, Beijing, China

**Keywords:** subtype, entosis, EML staining, multiplex staining, prognosis, cell-in-cell structures, esophageal squamous cell carcinoma, functional pathology

## Abstract

Cell-in-cell (CIC) structures are defined as the special structures with one or more cells enclosed inside another one. Increasing data indicated that CIC structures were functional surrogates of complicated cell behaviors and prognosis predictor in heterogeneous cancers. However, the CIC structure profiling and its prognostic value have not been reported in human esophageal squamous cell Carcinoma (ESCC). We conducted the analysis of subtyped CIC-based profiling in ESCC using “epithelium-macrophage-leukocyte” (EML) multiplex staining and examined the prognostic value of CIC structure profiling through Kaplan-Meier plotting and Cox regression model. Totally, five CIC structure subtypes were identified in ESCC tissue and the majority of them was homotypic CIC (hoCIC) with tumor cells inside tumor cells (TiT). By univariate and multivariate analyses, TiT was shown to be an independent prognostic factor for resectable ESCC, and patients with higher density of TiT tended to have longer post-operational survival time. Furthermore, in subpopulation analysis stratified by TNM stage, high TiT density was associated with longer overall survival (OS) in patients of TNM stages III and IV as compared with patients with low TiT density (mean OS: 51 *vs* 15 months, *P* = 0.04) and T3 stage (mean OS: 57 *vs* 17 months, *P*=0.024). Together, we reported the first CIC structure profiling in ESCC and explored the prognostic value of subtyped CIC structures, which supported the notion that functional pathology with CIC structure profiling is an emerging prognostic factor for human cancers, such as ESCC.

## Introduction

Esophageal cancer ranks the top 10 among most deadly cancers. It has two major histological types: adenocarcinoma (EAC) and squamous cell carcinoma (ESCC) (1). In China, over 90% of the cases of esophageal cancer are ESCC, which is the fourth most prevalent cancer of the country. ESCC is a highly aggressive malignancy with poor prognosis. However, even the esophageal cancers that belong to same stage are variable in terms of recurrence, mortality rates, and disease prognosis. Therefore, further exploration of mechanism underlying heterogeneity and identifying novel prognostic factor is needed (2–6).

Cell-in-cell (CIC) structures are defined as the special structures with one or more cells enclosed inside another one and prevalent in a wide range of human cancers (7). In cancer tissues, CIC structures could form homotypically between tumor cells or heterotypically between different types of cells, such as tumor cell and immune cells (8). The profiling of CIC structures indicated the direct interaction between tumor cells, and immune cell in micro-environment could promote the formation of CIC structures that lead to the death of the internalized cells. An increasing number of studies have suggested that more CIC structures were associated with poor prognosis in cancers and reports on CIC structure profiling as functional pathological surrogate of complicated cell behaviors and prognostic predictor in specific cancers are being reported (9–14).

In this study, we explored the CIC structure profiling in ESCC tissues by using the EML method established previously (15), and the association between CIC subtypes and prognosis of esophageal cancer was analyzed.

## Materials and Methods

### Patients and Samples

We performed a retrospective analysis of patients with histologically confirmed ESCC after esophagectomy. This study consisted of 71 samples (whole slides) of ESCC patients, who were treated between February 2014 and March 2019 at the Fifth Medical Center of Chinese PLA General Hospital (Beijing, China) and the Second Affiliated Hospital of Air Force Military Medical University (Xian, China), and 70 samples of ESCC patients from a tumor microarray (TMA) purchased from Shanghai Outdo Biotech Co. Ltd (Heso-Squ172Sur-01, XT11-020). The tissue microarray spotted with 172 tissue cores with a diameter of 1.5 mm, consisting of 94 esophageal cancer samples and 78 samples from adjacent esophageal cancer tissue. Finally, 70 esophageal cancer samples in the tissue microarray were involved in the final analysis based on pathological characteristic selection. Informed written consent was obtained from the patients. The study was approved by the institutional ethical committee of the Fifth Medical Center and Second Affiliated Hospital of Air Force Military Medical University. Patients that exhibited other types of malignancy or had succumbed to illness within 1 month post the surgery were excluded from the study. Follow-up data were available for all 141 patients, ranging between 1 and 85 months post the surgery (mean, 29.96 months).

### Immunostaining and Image Processing of Tissue Samples

Totally, 141 tissue samples of ESCC were collected for immunostaining. In addition, 10 paired para-carcinoma tissue and 80 normal tissues were used as control, respectively. The thickness of each tissue section is 4 μm. “EML method” was used to subtype CIC structure as previously reported (15), with antibodies against E-cadherin for epithelium, CD68 for macrophage, and CD45 for leukocyte. In brief, samples were first stained with antibody against CD45 (mouse mAb from Boster, BM0091) at dilution of 1:400 by Opal Multiplex tissue staining kit (Perkin Elmer, NEL791001KT) according to the standard protocol provided, which was eventually labeled with cyanine 3 fluorophore. Slides were then incubated with mixed antibodies against CD68 (rabbit pAb from Proteintech, 25747–1-AP) and E-cadherin (mouse mAb from BD Biosciences, 610181), followed by secondary antibodies of Alexa Fluor 647 anti-rabbit antibody (Invitrogen, A21245) and Alexa Fluor 488 anti-mouse antibody (Invitrogen, A11029), respectively. Fluorescence exited at the 488- and 647-nm wavelength was in spectrum similar to FITC and Cy5. Samples were also co-stained with antibodies against Caveolin-1 (rabbit mAb Cell Signaling Technology, 2267) and Ezrin (BD Biosciences, 610602), labeled with Cy5 and Cy3, respectively, as well as LAMP3 (Santa Cruz, sc-5275), labeled with Cy5. All slides were counterstained with DAPI to show nuclei and mounted with antifade reagent (Invitrogen, Carlsbad, CA). Multispectral images were taken with TMA modules of Vectra^®^ Automated Imaging System (Akoya) by 20× objective lens. Nuance system (Akoya) was used to build libraries of each spectrum (DAPI, FITC, Cy5, and Cy3) and unmix multispectral images with autofluorescence subtracted in high contrast and accuracy. InForm automated image analysis software package (Akoya) was used for batch analysis of multispectral images based on specified algorithms.

### CIC Structure Profiling and Quantification

Cellular structure where one or more cells morphologically fully enclosed by another cell with crescent nucleus was scored as CIC structures. Cell boundary of epithelial cells could be told by E-cadherin, which labels cell membrane. Additionally, CD45 and CD68 were applied to label cell body of leukocytes and macrophages, respectively. According to the expression feature of these three markers, we defined five different CIC structure subtypes in ESCC tissues, including (A) tumor cell in tumor cell (TiT, both cells expressed E-cadherin but negatively for CD45 and CD68), (B) leucocyte in tumor (LiT, inner cell is only positive for CD45, while outer cell only express E-cadherin), (C) tumor in macrophage (TiM, inner cell is only positive for E-cadherin, while outer cell only express CD68), (D) leukocytes in macrophages (LiM, inner cell is only positive for CD45, while outer cell only express CD68), and (E) macrophage in tumor (MIT, inner cell is only positive for CD68, while outer cell only express E-cadherin).

Only those structures with inner cells morphologically fully enclosed were counted. CIC structure subtypes were defined based on the types of cell involved: TiT for E-cadherin^+^ cells in E-cadherin^+^ cells (Tumor cells in Tumor cells); TiM for E-cadherin^+^ cells in CD68^+^ cells (Tumor cells in Macrophages); MiT for CD68^+^ cells in E-cadherin^+^ cells (Macrophages in Tumor cells); LiT for CD45^+^ cells in E-cadherin^+^ cells (Leukocytes in Tumor cells); LiM for CD45^+^ cells in CD68^+^ cells (Leukocytes in Macrophages). Overall CIC structures (oCICs) indicated the total of all kinds of CIC structures. CIC structure density in tissue was calculated as CIC structure number per mm^2^. Double blind reviews were performed by three experienced investigators in the quantification of CIC structure subtypes.

### Statistical Analysis

OS duration was defined as time from the date of surgery to death or to the most recent contact or visit. Associations between CIC structure counts and the clinicopathological characteristics of the patients were analyzed using the Spearman rank test. Proportional hazard assumption was checked by both graphically and hypothetically using a hypothesis test called Shoenfield residual test for the oCIC and TiT variables. Results showed that Shoenfield residuals were not associated with the time (*p* > 0.05), suggesting that this model satisfies the proportional hazards assumption (**Figure S2**). The continuous variables were dichotomized for OS using the “surv_cutpoint” function of the “survminer” R package, which determine the optimal cutpoints to separate high and low cell-in-cell groups at once based on maximally selected rank statistics (16, 17). The standardized log-rank statistics across all the cutoffs were shown to depict the selection of optimal cutpoints (**Figure S3**). Survival curves were plotted using the Kaplan-Meier method, and the differences in survival distributions between two groups were compared by the log-rank test. Cox univariate and multivariate regression analyses were conducted to determine the factors that were independently associated with patients’ OS. All statistical analyses were performed using SPSS 24.0 soft-ware (IBM, Armonk, NY, USA) and GraphPad Prism 5.0. For all these analyses, a *P* value less than 0.05 was considered statistically significant.

## Results

### Patient Characteristics

Totally, the data of 141 patients with ESCC were analyzed in this retrospective study. The patient characteristics were shown in **Table 1** and **Table S1**. The median age was 64.0 years (range, 31–81 years), and most patients were male (79.4%). The most common locations of lesion were middle esophagus (39.0%) and lower esophagus (34.0%). None of patients had received preoperative chemotherapy and radiotherapy.

**Table 1:** Association of CIC subtypes with clinical pathological characteristics.

		TiT			TiM				LiM			LiT				MiT				HeCICs				oCICs			
	N (%)	Low n (%)	High n (%)	*P*	Yes^#^ n (%)		No^#^ n (%)	*P*	Yes^#^ n (%)	No^#^ n (%)	*P*	Yes^#^ n (%)	No^#^ n (%)		*P*		Yes^#^ n (%)	No^#^ n (%)	*P*		Low n (%)	High n (%)	*P*	Low n (%)	High n (%)	*P*
Total	141	122 (86.5)	19 (13.5)		29 (20.6)		112 (79.4)		9 (6.4)	132 (93.6)		39 (27.7)	102 (72.3)				33 (23.4)	108 (76.6)			96 (68.1)	45 (31.9)		117 (83.0)	24 (17.0)	
Age (years)				0.783				0.875			0.525				0.835				0.217				0.754			0.887
≤60	46 (32.6)	40 (87.0)	6 (13.0)		8 (17.4)		38 (82.6)		3 (6.5)	43 (93.5)		14 (30.4)	32 (69.6)				9 (19.6)	37 (80.4)			34 (73.9)	12 (26.1)		40 (87.0)	6 (13.0)	
60-70	57 (40.4)	48 (84.2)	9 (15.8)		13 (22.8)		44 (77.2)		5 (8.8)	52 (91.2)		14 (24.6)	43 (75.4)				12 (21.1)	45 (78.9)			36 (63.2)	21 (36.8)		44 (77.2)	13 (22.8)	
>70	38 (27.0)	34 (89.5)	4 (10.5)		8 (21.1)		30 (78.9)		1 (2.6)	37 (97.4)		11 (28.9)	27 (71.1)				12 (31.6)	26 (68.4)			26 (68.4)	12 (31.6)		33 (86.8)	5 (13.2)	
Sex				0.583				0.607			0.116				0.35				0.701				0.307			0.607
Male	112 (79.4)	96 (85.7)	16 (14.3)		23 (20.5)		89 (79.5)		9 (8.0)	103 (92.0)		33 (29.5)	79 (70.5)				27 (24.1)	85 (75.9)			73 (65.2)	39 (34.8)		92 (82.1)	20 (17.9)	
Female	29 (20.6)	26 (89.7)	3 (10.3)		6 (20.7)		23 (79.3)		0 (0.0)	29 (100.0)		6 (20.7)	23 (79.3)				6 (20.7)	23 (79.3)			23 (79.3)	6 (20.7)		25 (86.2)	4 (13.8)	
Location				0.214				0.773			**0.014**				0.349				0.38				0.797			0.129
Upper	10 (7.1)	8 (80.0)	2 (20.0)		1 (10.0)		9 (90.0)		0 (0.0)	10 (100.0)		4 (40.0)	6 (60.0)				1 (10.0)	9 (90.0)			6 (60.0)	4 (40.0)		7 (77.8)	2 (22.2)	
Middle	55 (39.0)	48 (87.3)	7 (12.7)		12 (21.8)		43 (78.2)		2 (3.6)	53 (96.4)		12 (21.8)	43 (78.2)				13 (23.6)	42 (76.4)			36 (65.5)	19 (34.5)		45 (81.8)	10 (18.2)	
Lower	50 (35.5)	40 (80.0)	10 (20.0)		13 (26.0)		37 (74.0)		2 (4.0)	48 (96.0)		11 (22.0)	39 (78.0)				11 (22.0)	39 (78.0)			35 (70.0)	15 (30.0)		36 (75.0)	12 (25.0)	
Unknown	26 (18.4)	26 (100.0)	0 (0.0)		3 (11.5)		23 (88.5)		5 (19.2)	21 (80.8)		12 (46.2)	14 (53.8)				8 (30.8)	18 (69.2)			19 (73.1)	7 (26.9)		29 (100.0)	0 (0.0)	
TNM stage				0.353				0.431			0.406				0.639				0.827				0.398			0.917
I+II	75 (53.2)	63 (84.0)	12 (16.0)		13 (17.3)		62 (82.7)		6 (8.0)	69 (92.0)		22 (29.3)	53 (70.7)				17 (22.7)	58 (77.3)			52 (69.3)	23 (30.7)		62 (82.7)	13 (17.3)	
III+IV	66 (46.8)	59 (89.4)	7 (10.6)		16 (24.2)		50 (75.8)		3 (4.5)	63 (95.5)		17 (25.8)	49 (74.2)				16 (24.2)	50 (75.8)			44 (66.7)	22 (33.3)		55 (83.3)	11 (16.7)	
T stage				0.188				0.863			0.257				0.989				0.277				0.468			0.630
T1	8 (5.7)	8 (100.0)	0 (0.0)		1 (12.5)		7 (87.5)		1 (12.5)	7 (87.5)		1 (12.5)	7 (87.5)				1 (12.5)	7 (87.5)			7 (87.5)	1 (12.5)		8 (100.0)	0 (0.0)	
T2	23 (16.3)	17 (73.9)	6 (26.1)		4 (17.4)		19 (82.6)		2 (8.7)	21 (91.3)		6 (26.1)	17 (73.9)				3 (13.0)	20 (87.0)			16 (69.6)	7 (30.4)		17 (73.9)	6 (26.1)	
T3	99 (70.2)	86 (86.9)	13 (13.1)		22 (22.2)		77 (77.8)		6 (6.1)	93 (93.9)		31 (31.3)	68 (68.7)				27 (27.3)	72 (72.7)			64 (64.6)	35 (35.4)		82 (82.8)	17 (17.2)	
T4	11 (7.8)	11 (100.0)	0 (0.0)		2 (18.2)		9 (81.8)		0 (0.0)	11 (100.0)		1 (9.1)	10 (90.9)				2 (18.2)	9 (81.8)			9 (81.8)	2 (18.2)		10 (90.9)	1 (9.1)	
N stage				0.256				0.55			0.301				0.653				0.947				0.413			0.791
N0	71 (50.4)	59 (83.1)	12 (16.9)		13 (18.3)		58 (81.7)		6 (8.5)	65 (91.5)		22 (31.0)	49 (69.0)				17 (23.9)	54 (76.1)			48 (67.6)	23 (32.4)		58 (81.7)	13 (18.3)	
N1	43 (30.5)	39 (90.7)	4 (9.3)		8 (18.6)		35 (81.4)		2 (4.7)	41 (95.3)		8 (18.6)	35 (81.4)				9 (20.9)	34 (79.1)			32 (74.40)	11 (25.6)		37 (86.0)	6 (14.0)	
N2	19 (13.5)	16 (84.2)	3 (15.8)		6 (31.6)		13 (68.4)		1 (5.3)	18 (94.7)		7 (36.8)	12 (63.2)				6 (31.6)	13 (68.4)			10 (52.6)	9 (47.4)		15 (78.9)	4 (21.1)	
N3	8 (5.7)	8 (100.0)	0 (0.0)		2 (25.0)		6 (75.0)		0 (0.0)	8 (100.0)		2 (25.0)	6 (75.0)				1 (12.5)	7 (87.5)			6 (75.0)	2 (25.0)		7 (87.5)	1 (12.5)	
Histology grade				0.72				0.492			0.76				0.818				0.127				0.438			0.813
I	10 (7.1)	8 (80.0)	2 (20.0)		3 (30.0)		7 (70.0)		0 (0.0)	10 (100.0)		4 (40.0)	6 (60.0)				3 (30.0)	7 (70.0)			6 (60.0)	4 (40.0)		8 (80.0)	2 (20.0)	
II	100 (70.9)	87 (87.0)	13 (13.0)		20 (20.0)		80 (80.0)		8 (8.0)	92 (92.0)		26 (26.0)	74 (74.0)				26 (26.0)	74 (74.0)			70 (70.0)	30 (30.0)		83 (83.0)	17 (17.0)	
III	31 (22.0)	27 (87.1)	4 (12.9)		6 (19.4)		25 (80.6)		1 (3.2)	30 (96.8)		9 (29.0)	22 (71.0)				4 (12.9)	27 (87.1)			20 (64.5)	11 (35.5)		26 (83.9)	5 (16.1)	

TiT Low: <3.721 CICs/mm^2^, High: ≥3.721 CICs/mm^2^; HeCICs Low: <0.625 CICs/mm^2^, High: ≥0.625 CICs/mm^2^; oCICs Low: <4.651 CICs/mm^2^, High: ≥4.651 CICs/mm^2^; No: 0 CICs/mm^2^; Yes: ≥0 CICs/mm^2^. Spearman rank test was used to determine the association between variables.

In bold: P value less than 0.05.

### CIC Profiling in ESCC

**Figure 1:**
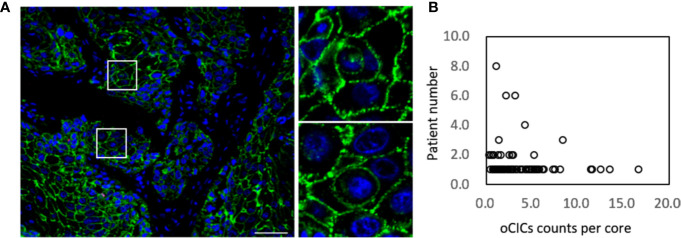
Cell-in-cell structures in esophagus carcinoma. **(A)** Representative image for CICs in human esophagus carcinoma co-stained with antibodies for E-cadherin, CD45, CD68 and DAPI. Right panels show zoomed images for boxed regions in the left image. Scale bar: 100 μm. **(B)** Distribution of overall CICs (oCICs) across esophagus carcinoma tissues from different patients.

Totally, 141 ESCC tissue samples, 10 paired para-carcinoma tissues and 80 paired normal tissues were stained by using the EML method. CIC structures were positive in 121 tumor tissue with the average density as 2.5 CIC/mm² (range, 0.5–24.0 CIC/mm²; **Figure 1**). However, there was no CIC detected in para-carcinoma or normal esophagus tissue.

**Figure 2:**
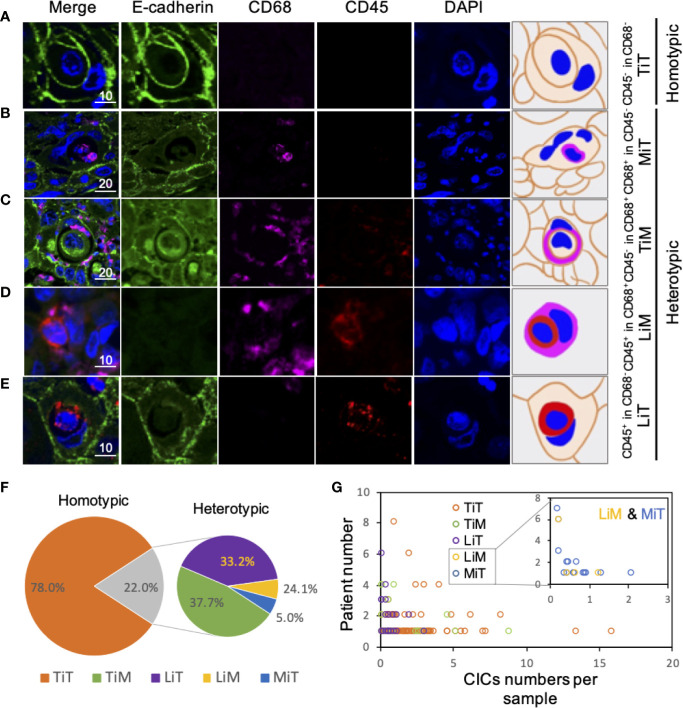
Subtype profiling of cell-in-cell structures in esophagus cancer. **(A–E)** Representative images for five CIC subtypes as indicated. Right panels of pictures demonstrate the schematic structure for each CIC subtype. Scale bar: 10 or 20 μm. **(F)** Distribution of five CIC subtypes across esophagus cancer tissues in all patients. **(G)** Distribution of five CIC subtypes in esophagus cancer tissues from different patients.

We exploited three molecules, E-cadherin for epithelial cell membrane, CD45 for the leukocytes, and CD68 for the macrophages to identify CIC subtypes. Based on the “epithelium-macrophage-leukocyte” (EML) multiplex staining method, we defined five different CIC structure subtypes in ESCC tissues including (A) tumor cell in tumor cell (TiT), (B) leucocyte in tumor (LiT), (C) tumor in macrophage (TiM), (D) leukocytes in macrophages (LiM), and (E) macrophage in tumor (MIT) (**Figures 2A–E**). TiT was also named with homotypic CIC (HoCIC) structures, and the other four subtypes of CIC structures were summarized as heterotypic CIC (HeCIC) structures. Besides, molecules, like Caveolin-1, Ezrin, and LAMP3, were also checked in esophageal carcinoma tissues. The result showed that the positive rate of Ezrin in HoCIC structure was higher than that of caveolin-1, indicating a more active role of Ezrin in CIC structures of ESCC tissues. Interestingly, though LAMP3 expressed well in most of the ESCC tumor cells, only a small portion of CIC structures was positive in LAMP3, probably suggesting a low level of lysosomal activity in these CIC structures (**Figure S1**).

Among the subtypes of CIC structures, TiT was most prevalent subtype, which accounted for 78.0% of the total number of oCIC structures, and 97.5% (118) of oCIC-positive tumor tissue was TiT positive. As for HeCIC structures, TiM had the largest number, accounting for 37.7% of the total HeCIC structures, and 8.3% of oCIC structures; LiT and MiT ranked the second and third, accounting for 33.1% and 24.1% of the total HeCIC, 7.3% and 5.3% of oCIC structures, respectively; LiM was rare and accounted for 5.1% of the total HeCICs (**Figure 2F**).

### Association Between CIC Structure Subtypes and Clinicopathological Characteristics

To quantify the CIC structures more accurately, CIC density, for CIC structure counts per mm^2^, was introduced in this study. The CIC density in the 141 samples was shown in **Figure 2G**. Considering that majority of the CIC structures was HoCIC (or TiT) and HeCIC quantity was rather low, only oCIC structures, HeCIC structures, and TiTs were evaluated by density in subsequent analysis. The patients were divided into two groups according to optional cutpoint of CIC structures density based on the maximally selected rank statistics. Take oCIC structures as an example, the patients with density < 4.651 CIC structures/mm² were categorized as low oCIC structure group, while those with density ≥ 4.651 CIC structures/mm² were categorized as high CIC structure group. As such, the patients were dichotomize based on the optional cutpoints of HeCIC structures (0.625 CIC structures/mm²) and TiT (3.721 CIC structures/mm²) (**Figure S3**). For TiM, LiM, LiT, and MiT, the dichotomy was applied based on the presence and absence of corresponding CIC structure owing to the scarce of these CIC structure subtypes.

The association between CIC structure subtypes and the clinical information of patients was further analyzed, as shown in **Table 1**. The result indicated that, compared with the samples of upper ESCC, there were more LiM detected in the samples of lower ESCC (0% *vs* 50%, *P* = 0.014). CIC structure subtypes demonstrated no association with other traditional clinicopathological factors, including TNM stage, age, sex, and histology grade.

### Association Between CIC Structure Subtypes and Patients’ Survival

With the patients’ return visit, the survival information of 141 patients was collected, and the correlation between the levels of various CIC structure subtypes and survival time of the patients was analyzed. The results in **Table 2** showed that in esophagus cancer, the distribution of TiT was significantly different in patients with distinct survival duration. More narrowly, the proportion of more TiT was much higher in patients with survival duration 36 to 60 months than that in patients with survival duration less than 36 months or longer than 60 months.

**Table 2:** Association of CICs subtypes with survival characteristics.

		TiT			TiM			LiM			LiT			MiT			HeCICs			oCICs		
	N (%)	Low n (%)	High n (%)	*P*	Yes^#^ n (%)	No^#^ n (%)	*P*	Yes^#^ n (%)	No^#^ n (%)	*P*	Yes^#^ n (%)	No^#^ n (%)	*P*	Yes^#^ n (%)	No^#^ n (%)	*P*	Low n (%)	High n (%)	*P*	Low n (%)	High n (%)	*P*
Total	141	122 (86.5)	19 (13.5)		29 (20.6)	112 (79.4)		9 (6.4)	132 (93.6)		39 (27.7)	102 (72.3)		33 (23.4)	108 (76.6)		96 (68.1)	45 (31.9)		117 (83.0)	24 (17.0)	
Survival time (months)				**0.037**			0.759			0.212			0.135			0.139			0.835			0.168
<36	88 (62.4)	81 (92.0)	7 (8.0)		20 (22.7)	68 (77.3)		5 (5.7)	83 (94.3)		24 (27.3)	64 (72.7)		20 (22.7)	68 (77.3)		57 (64.8)	31 (35.2)		77 (87.5)	11 (12.5)	
≥36 and <60	32 (22.7)	22 (68.8)	10 (31.2)		5 (15.6)	27 (84.4)		2 (6.2)	30 (93.8)		9 (28.1)	23 (71.9)		8 (25.0)	24 (75.0)		21 (65.6)	11 (34.4)		22 (68.8)	10 (31.2)	
≥60	21 (14.9)	19 (90.5)	2 (9.5)		4 (19.0)	17 (81.0)		2 (9.5)	19 (90.5)		6 (28.6)	15 (71.4)		5 (23.8)	16 (76.2)		18 (85.7)	3 (14.3)		18 (85.7)	3 (14.3)	

TiT low, <3.721 CICs/mm^2^; high, ≥3.721 CICs/mm^2^; HeCICs low, <0.625 CICs/mm^2^; high, ≥0.625 CICs/mm^2^; oCICs low, <4.651 CICs/mm^2^; high, ≥4.651 CICs/mm^2^; No: 0 CICs/mm^2^; yes, ≥0 CICs/mm^2^. Spearman rank test was used to determine the association between variables.

In bold: P value less than 0.05.

The relation between CIC structures and patients’ survival was then assessed by Cox regression model. In univariate analysis, TiT density, TNM stage, T stage, and N stage was significantly correlated with the OS of ESCC patients, respectively, as shown in **Table 3** (HR: 0.48249882, *P* = 0.028905; HR: 2.45798584, *P* = 0.000008; HR: 1.76727404, *P* = 0.000513; HR: 1.57737502, *P* = 0.000022).

**Table 3:** Association of overall survival with clinicopathological parameters and CICs by univariate Cox regression analysis.

characteristics		n	HR	95% CI	*P*
TiT	High	19	0.48249882	0.251–0.928	**0.028905**
	Low	122			
TiM	Yes	29	1.17542548	0.739–1.869	0.494450
	No	112			
LiM	Yes	9	0.61991250	0.272–1.415	0.256278
	No	132			
LiT	Yes	39	1.02780496	0.675–1.565	0.898302
	No	102			
MiT	Yes	33	1.16645531	0.748–1.819	0.497203
	No	108			
HeCICs	High	45	1.22446452	0.817–1.835	0.326699
	Low	96			
oCICs	High	24	1.56482122	0.904–2.709	0.109844
	Low	117			
Age	≤60	46	1.02248339	0.797–1.312	0.861220
	60-70	57			
	>70	38			
Sex	Male	112	0.65058457	0.391–1.082	0.097650
	Female	29			
Location	Upper	10	0.97985858	0.776–1.238	0.864453
	Middle	55			
	Lower	50			
	Unknown	26			
TNM stage	I+II	75	2.45798584	1.656–3.648	**0.000008**
	III+IV	66			
T stage	T1	8	1.76727404	1.282–2.437	**0.000513**
	T2	23			
	T3	99			
	T4	11
N stage	N1	71	1.57737502	1.278–1.947	**0.000022**
	N2	43			
	N3	19			
	N4	8			
Histology grade	I	10	0.91238536	0.630–1.322	0.628054
	II	100			
	III	31			

TiT low, <3.721 CICs/mm^2^; high, ≥3.721 CICs/mm^2^; HeCICs low, <0.625 CICs/mm^2^; high, ≥0.625 CICs/mm^2^; oCICs low, <4.651 CICs/mm^2^; high, ≥4.651 CICs/mm^2^; no, 0 CICs/mm^2^; yes, ≥0 CICs/mm^2^.

In bold: P value less than 0.05.

**Figure 3:**
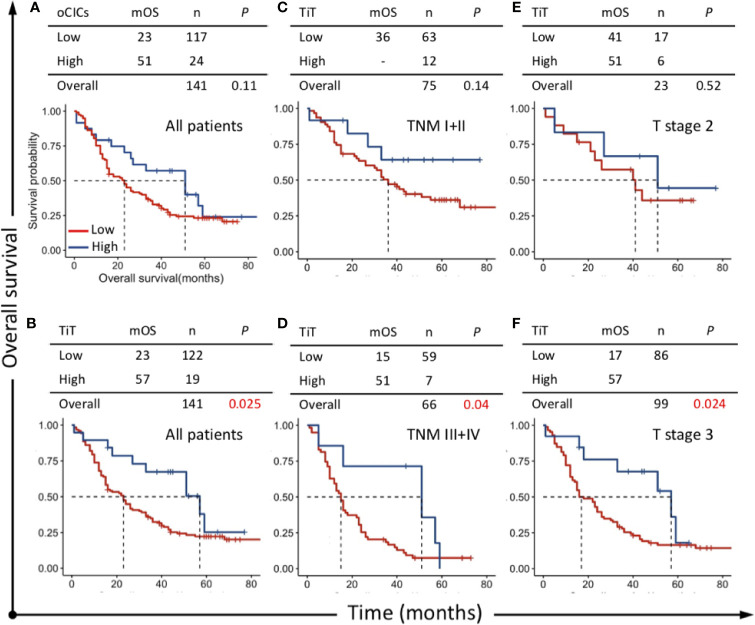
Survival impacts of CICs on overall survival (OS) of esophagus cancer patients. Kaplan–Meier plotting for OS curves of **(A)** oCICs, **(B)** TiT, and **(C–F)** TiT in stratified patients with different TNM and T stages. TiT Low: <3.721 CICs/mm^2^, High: ≥3.721 CICs/mm^2^; oCICs Low: <4.651 CICs/mm^2^, High: ≥4.651 CICs/mm^2^.

Survival curve was plotted by Kaplan-Meier method, and the difference was assessed by log-rank test. The results indicated that patients with high TiT density showed a favorable prognosis and longer median OS (mOS: 57 *vs* 23 months, *P*=0.025) (**Figure 3B**) compared with patients with low TiT density. However, oCIC structures failed to show any association with survival time (**Figures 3A, B**).

### CIC Structures Are Independent Prognostic Factor for Esophagus Cancer

To analyze whether CIC structures and their subtypes are independent prognostic factors for postoperative survival of ESCC patients, all the variables identified in univariate analysis (TiT density, TNM stage, T stage and N stage) were then included in further multivariate analysis using Cox proportional hazards model. The result indicated that TiT density (HR = 0.490, *P* = 0.034) and T stage (HR = 1.451, *P* = 0.040) were independent prognostic factors for ESCC patients (**Table 4**). In detail, more TiT in tumor tissue and early T stage could decrease the death risk of ESCC patients by nearly 50%.

**Table 4:** Multivariate Cox regression analysis of overall survival.

Variables	n	HR	*P*
TiT		0.490 (0.253–0.948)	**0.034**
Low	122		
High	19		
TNM stage		1.506 (0.790–2.869)	0.213
I+II	75		
III+IV	66		
T stage		1.451 (1.017–2.072)	**0.040**
T1	8		
T2	23		
T3	99		
T4	11		
N stage		1.247 (0.898–1.731)	0.188
N0	71		
N1	43		
N2	19		
N3	8		

TiT low, <3.721 CICs/mm^2^; high, ≥3.721 CICs/mm^2^.

In bold: P value less than 0.05.

### Homotypic CIC Structures Preferentially Impact the Survival of Patients of Late TNM Stage

To clarify the prognostic value of TiT in different subpopulation of ESCC patients, the correlation of TiT density and OS was analyzed when the patients were stratified by different TNM stage or T stage. The result indicated that high TiT density was associated with longer OS in patients of TNM stage III and IV as compared with patients with low TiT density (mOS: 51 *vs* 15 months, *P* = 0.04) and T3 stage (mOS: 57 *vs* 17 months, *P*=0.024). Meanwhile, in patients with early stage such as TNM stage I and II, there was no difference in prognosis between high and low TiT density groups, as well as T2 stage (**Figures 3C–F**). These results suggested that TiT density may potentially have better prognostic value in patients of later stage or higher risks; however, it should be validated in further study with amplified patient size and in a prospective way.

## Discussion

In this study, we examined the CIC profiles of ESCC and paired adjacent normal tissues and identified five CIC subtypes in ESCC tissues. Among the five subtypes, TiT served as an independent prognostic factor. On top of that, TiT tended to demonstrate better prognostic performance in patients of rather later TNM stage or T stage (*P* = 0.04 or 0.024), which warrants further validation in future studies.

CIC structure is a special way of cell death with high environmental specificity that kills the internalized cells generally in an acidified lysosome-dependent way (8, 18). Cell death caused by CICs is mediated by three core elements including adherens junction, contractile actomyosin and mechanical ring as well (19–21). Besides, CIC structure formation is also regulated by a set of factors, such as CDKN2a (22), PCDH7 (23), IL8 (24) and membrane lipids (25). Functionally, CIC structure-mediated non-autonomous inner cell death is conducive to cell competition in mammals (7, 26–28). Therefore, CIC structures are also regarded as a way for tissues to maintain homeostasis (29, 30). Disorder in this process may lead to tumors or diseases such as immune-related disorders (8, 31–33). At present, literatures have reported CIC structures in tumor tissues, including urothelial carcinoma (34), buccal mucosa squamous cell carcinoma (10), pancreatic ductal adenocarcinoma (9), metastatic adenocarcinoma (34), head and neck squamous cell carcinoma (35), renal cell carcinoma (36), gastric cancer (37), breast carcinoma (11, 38), small cell carcinoma of lung (39), benign tendon sheath giant cell carcinoma specimen (40), malignant mesothelioma (41), leiomyolipoma (42), etc., but there is no study on the CIC structures in the tissues of esophageal cancer.

Current determination of CIC structures was mainly based on the cellular morphology that was readout by ways of tissue staining. Hematoxylin-Eosin (H&E) staining and May-Grunwald-Giemsa (MGG) staining were two usual methods to distinguish cell nucleus and membrane. Papanicolaou staining was used for polychromatic staining, of which the section transparency is better, and the cytoplasm in a variety of cells present different colors, which is convenient for determining cell types. Similarly, EML staining can also help to distinguish cell types, and more precisely, in which epithelial cells, macrophages and leukocytes were marked by epithelial cadherin, CD68, and CD45, respectively. The results showed that the 5 subtypes reported in other cancers were also detected in human ECSS tissues, of which TiT was much higher than the other 4 subtypes. In addition, the density of subtyped CIC structures are correlated with location and prognosis of ESCC, suggesting that CIC structures may be a candidate marker for clinical diagnosis of esophagus cancer. There may also be other types of cell cannibalism, which requires screening with more cellular markers.

As for the level of CIC structures in the previous studies, other quantitative methods were used. In urothelial carcinoma and metastatic adenocarcinoma, where the number of CIC structures in 100 tumor cells was set as CIC structure index (34); Except for this, in the experiment of breast ductal carcinoma, the number of CIC structures in 1000 tumor cells was also served as CIC structure index (43). To calculate CIC structure index, the total number of tumor cells, other than those in CIC structures, in a defined field shall be counted, which tended to produce more systemic errors. While in this paper, we used CIC structure density as the readout, which was calculated as CIC structure number in a defined area, so it is more suitable to clinical practices.

Previous experiments about melanoma cells showed that cell ingestion in cancer arises owing to the need for metabolism of individual cells, which increases intracellular nutrient pools in order to support cancer cell survival and proliferation (44). Similarly, the internalization of tumor cells may increase the instability of genome in target cells, thereby promoting the development of tumors in the long term. On the other hand, CIC formation as a mechanism of cell cannibalism that is induced by the establishment of epithelial adhesion could inhibit transformed growth, suggesting that CIC structure-mediated cell killing might also be a potential tumor suppressive pathway (7, 20). Cannibalistic behavior, as one of the mechanisms involving CIC formation, may increase nutrient intake by feeding upon other cells, and escape from the specific immune response by engulfing lymphocytes (33). In this scenario, upregulation of phagocytosis in human tumor cells may be similar to that of some unicellular microorganisms, of which the goal is to survive and propagate in a hostile microenvironment (8, 45). Moreover, while this study dealt with resectable cancer, increased CIC formation was also identified in metastatic cancer cells. For example, Lugini *et al.* found that the metastatic, but not the primary, melanoma cells displayed strong cannibalistic activity against live lymphocytes (44). This activity seems to be associated with the expression of TM9SF4, an important protein associated with phagocytic activity (45, 46), as well as v-ATPase, a master controller of vacuolar pH that is in complex with TM9SF4 to create a unique milieu favoring tumor metastasis and chemo/immune-resistance traits of solid tumors (47). Future investigations on the expressions and roles of TM9SF4 and v-ATPase in CIC structures of metastatic cancers would shed novel lights on the CIC structure formation by tumor cell cannibalism.

Together with classic clinicopathologic factors such as TNM stage, CIC subtype may provide more accurate information of prognosis prediction, especially in the situation where traditional prognostic factors have reached the ceiling roof. For ESCC patients with late TNM stage, low TiT could be helpful to identify patients with poorer outcomes who should be given more intensive treatment.

There were still several limitations in this study. First, the retrospective nature of this study may lead to bias inevitably, and well-designed prospective study will be needed. Second, there was no validation cohort in this study which should be considered in the future. Third, some samples came from the commercial TMA and the related treatment information including chemotherapy and radiotherapy were unavailable which may decrease the credibility of result to certain extents. Additionally, the quantification of CIC structures in tissues relays on multiple experienced investigators, which calls for an algorithm-based program for more standard and efficient quantification similar to that achieved recently on cytospins (48).

To sum up, we reported the CIC profiling in ESCC for the first time and preliminarily explored the prognostic value of CIC subtype in this study. TiT was identified as a potent prognostic marker in ESCC and showed more prognostic value in patients with late TNM stage or high risks. Our work also supports the notion that function pathology with CIC profiling is an emerging prognostic factor for human cancers.

## Data Availability Statement

The original contributions presented in the study are included in the article/**Supplementary Material**. Further inquiries can be directed to the corresponding authors.

## Ethics Statement

The studies involving human participants were reviewed and approved by The Outdo Biotech Co. Ltd., National Human Genetic Resources Sharing Service Platform. The patients/participants provided their written informed consent to participate in this study.

## Author Contributions

QS, HJ, and HH: concept and design. YW and ZN: staining and imaging. YW, ZN, LZ, YAZ, QM, YCZ, ML, YS, JG LG, and JS: data acquisition. QS, HH, and YW: manuscript drafting. YAZ, QM, QJS, and YT: pathological judgment and confirmation. YW and LZ: statistical analysis. All authors contributed to the article and approved the submitted version.

## Conflict of Interest

The authors declare that the research was conducted in the absence of any commercial or financial relationships that could be construed as a potential conflict of interest.
