# Supplementary figures and images for: Subtype-Based Analysis of Cell-in-Cell Structures in Esophageal Squamous Cell Carcinoma
Source: Front Oncol. 2021 Jun 11;11:670051. doi: 10.3389/fonc.2021.670051 (PMC8231019; doi:10.3389/fonc.2021.670051)

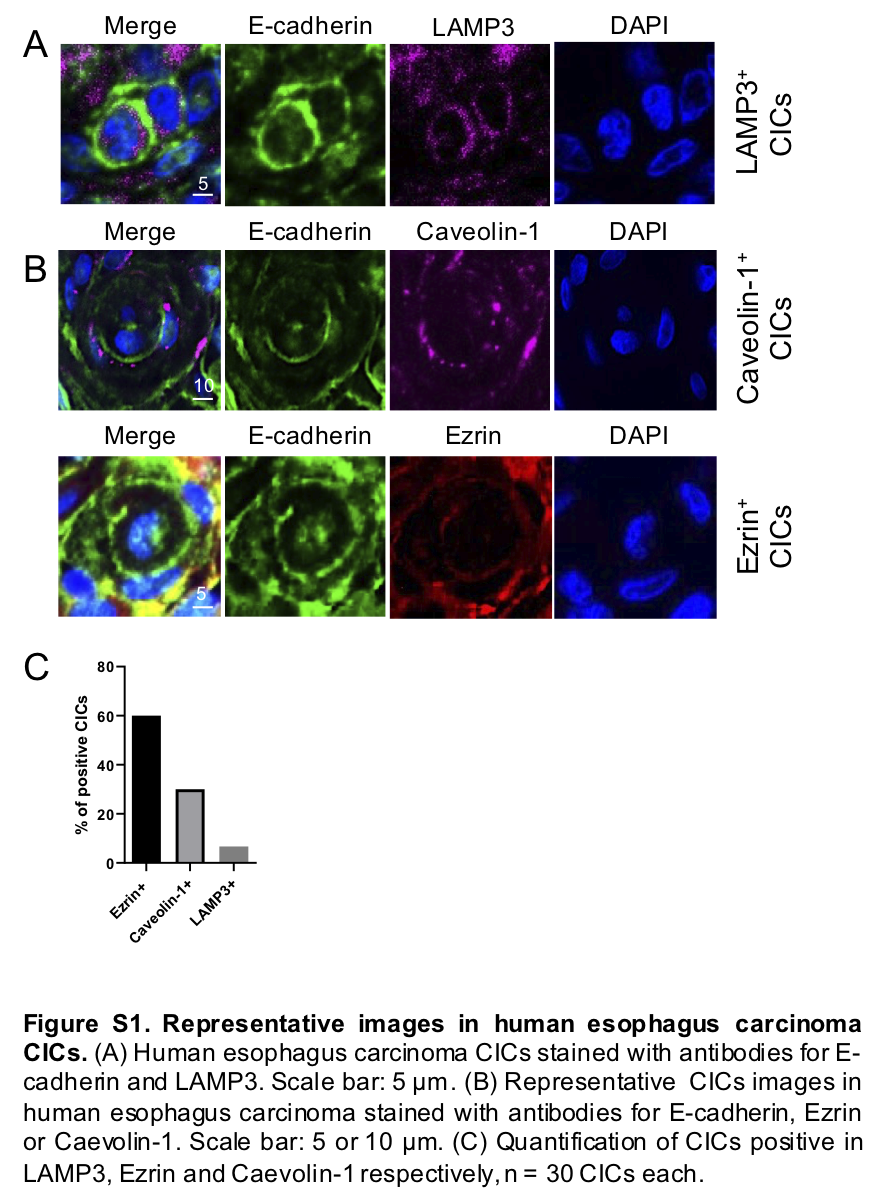

Supplement: Supplementary file 1 [file Image_1.tiff]

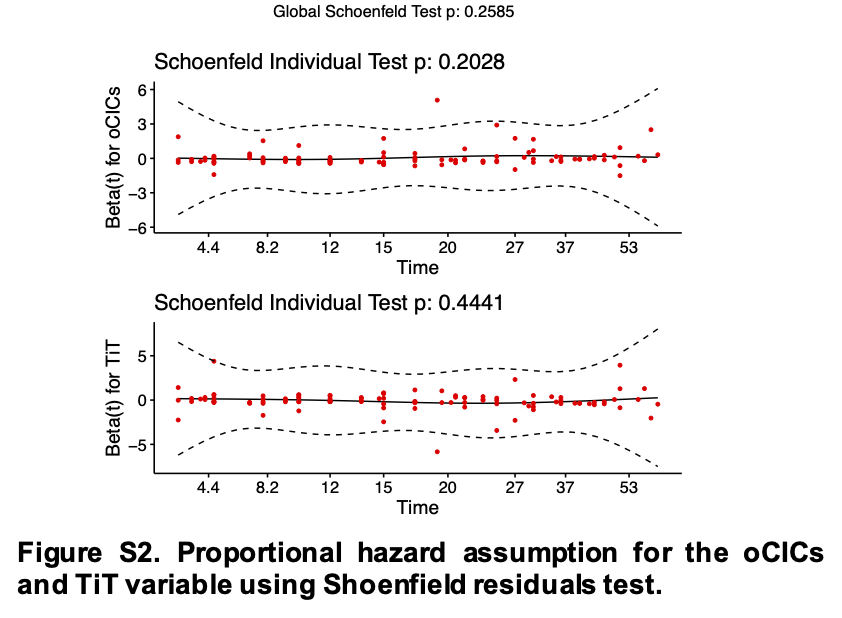

Supplement: Supplementary file 2 [file Image_2.tiff]

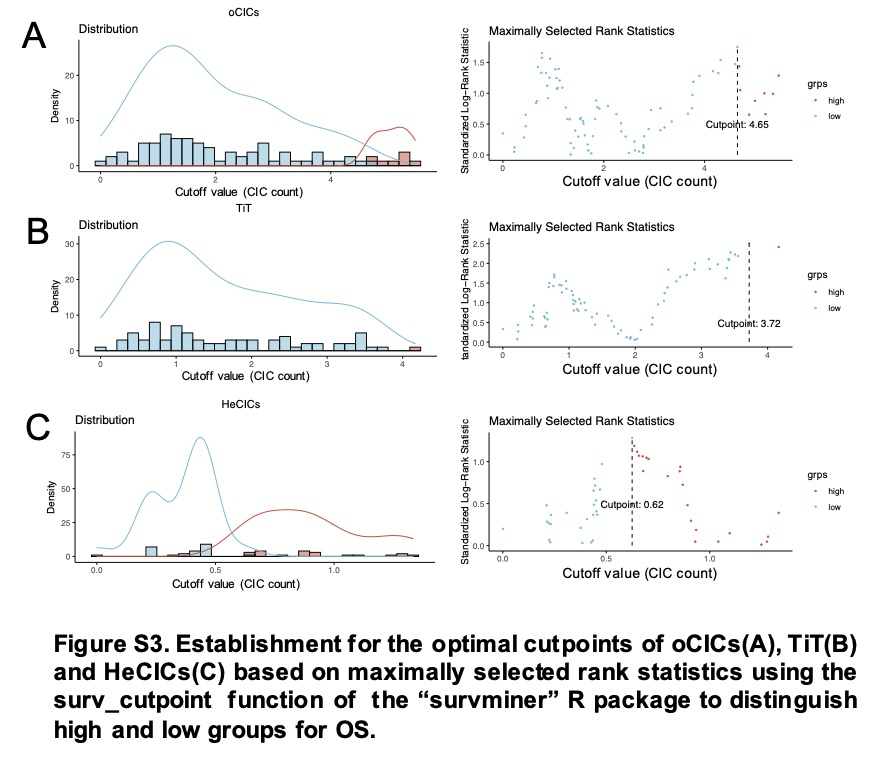

Supplement: Supplementary file 3 [file Image_3.tiff]
