# Supplementary material for: Subtype-Based Analysis of Cell-in-Cell Structures in Esophageal Squamous Cell Carcinoma
Source: Front Oncol. 2021 Jun 11;11:670051. doi: 10.3389/fonc.2021.670051 (PMC8231019; doi:10.3389/fonc.2021.670051)
Supplement: Supplementary file 4 [file Presentation_1.pptx]

## Slide 1
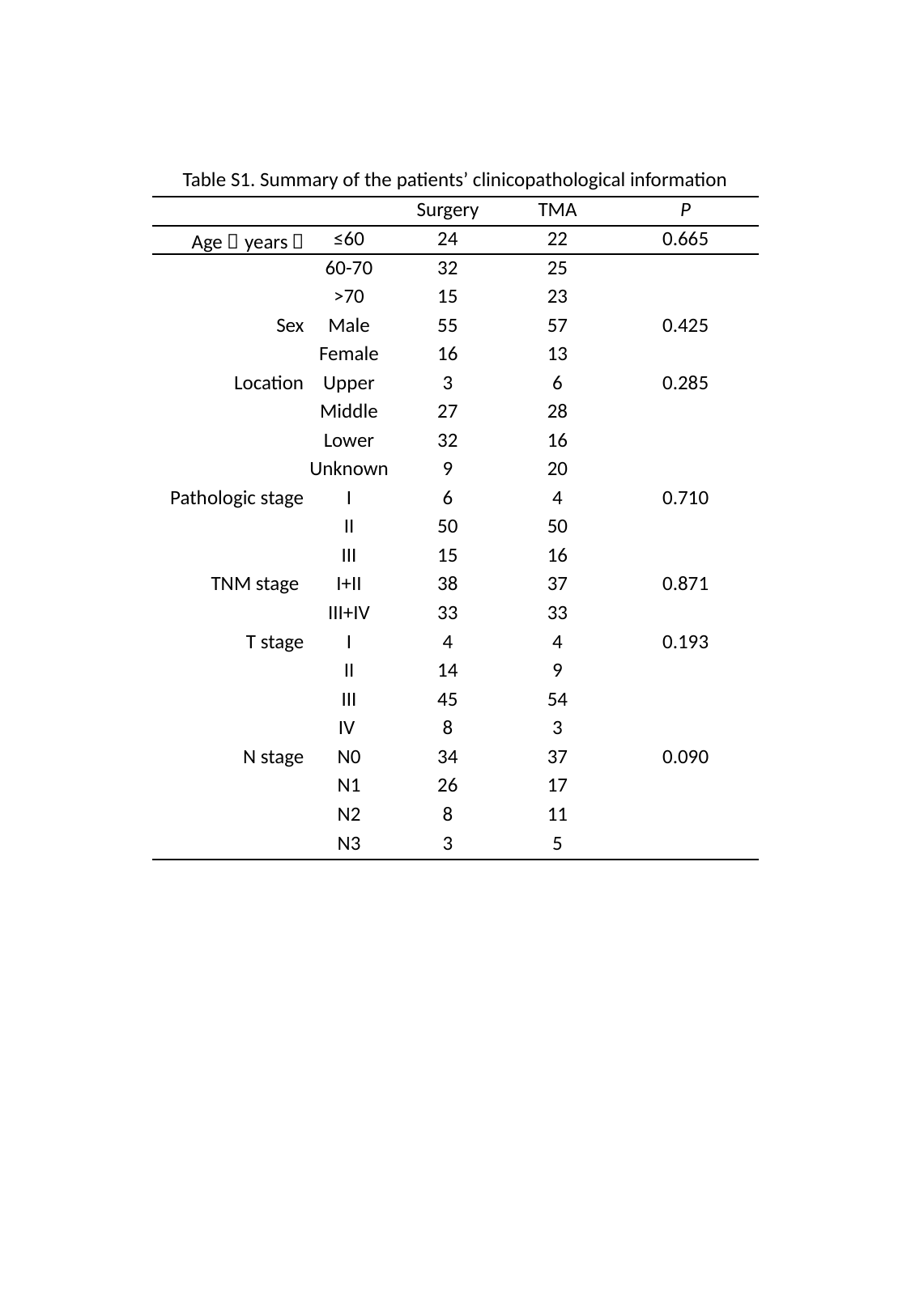

Table S1. Summary of the patients’ clinicopathological information
| | | Surgery | TMA | P |
| --- | --- | --- | --- | --- |
| Age（years） | ≤60 | 24 | 22 | 0.665 |
| | 60-70 | 32 | 25 | |
| | >70 | 15 | 23 | |
| Sex | Male | 55 | 57 | 0.425 |
| | Female | 16 | 13 | |
| Location | Upper | 3 | 6 | 0.285 |
| | Middle | 27 | 28 | |
| | Lower | 32 | 16 | |
| | Unknown | 9 | 20 | |
| Pathologic stage | I | 6 | 4 | 0.710 |
| | II | 50 | 50 | |
| | III | 15 | 16 | |
| TNM stage | I+II | 38 | 37 | 0.871 |
| | III+IV | 33 | 33 | |
| T stage | I | 4 | 4 | 0.193 |
| | II | 14 | 9 | |
| | III | 45 | 54 | |
| | IV | 8 | 3 | |
| N stage | N0 | 34 | 37 | 0.090 |
| | N1 | 26 | 17 | |
| | | | | |
| | N2 | 8 | 11 | |
| | | | | |
| | N3 | 3 | 5 | |
| | | | | |
